# Strain gauge analysis and deformation detection of maxillary denture bases reinforced by CAD/CAM milled zirconia and cobalt chromium (Co-Cr) frameworks: An *in-vitro* study

**DOI:** 10.1186/s12903-026-08069-0

**Published:** 2026-03-27

**Authors:** Maha Nagy Mohamed Kamal, Ghada Mahmoud Mansour ElGindy, Hebatallah Tarek Mohamed, Marwa Ezzat Sabet

**Affiliations:** 1 Faculty of Dentistry, British University in Egypt (BUE), Elshrouk city, Cairo Governorate Egypt; 2 Faculty of Dentistry, Ain Shams University, Abbassia city, Cairo Governorate Egypt

**Keywords:** Strain gauges, Denture base deformation, Maxillary denture, Zirconia framework, Cobalt chrome framework, CAD/CAM milling

## Abstract

**Background:**

A lot of studies have demonstrated the increased hardness of the reinforced maxillary dentures. However, the amount of stress induced by different reinforcement materials under the reinforcement dentures and their effect on the underlying supporting tissues and on the deformation of the denture bases were questionable. This study was conducted to compare the stresses induced within the supporting oral tissues from maxillary denture bases reinforced with computer-aided designing/computer-aided manufacturing CAD/CAM zirconia framework or cobalt chrome (Co-Cr) framework, and to detect the deformation that occurred to these bases.

**Materials and methods:**

Twenty-six maxillary complete dentures were fabricated and divided into two groups (n = 13). Group A (test group): zirconia framework reinforcement. Group B (control group): Co-Cr framework reinforcement. All denture bases were subjected to unilateral static loading, and then the strain was monitored using a strain gauge analysis device. Then the reinforced denture bases of both groups were subjected to dynamic cyclic loading, then deformation was detected using Geomagic software.

**Results:**

Group A; showed significantly higher strain measured at all measuring areas compared to Group B. The maximum principal strain (MPS) at the 1st right molar area was consistently the highest among all measuring areas, regardless of the denture reinforcement framework type. Deformation results suggested that Group A; showed statistically significant reduced deformation compared to Group B after dynamic cyclic load application.

**Conclusion:**

Zirconia framework-reinforced maxillary dentures were associated with higher strains and less framework deformation than cobalt chrome framework-reinforced maxillary dentures when static and dynamic loading were applied.

## Background 

Acrylic resin (polymethyl methacrylate) has been the most commonly used denture base material for over a century, despite its favorable properties such as excellent appearance, durability, simple processing, and ease of repair; however, acrylic resin has some disadvantages, such as low impact strength, water sorption, allergic reaction, and absence of adequate fatigue resistance [1].

Under stresses either intraorally from mastication or extra-orally from any impact forces, deformation and fracture of acrylic resin denture bases frequently occur, especially at the areas that are heavily stressed, such as lingual to the incisors, with the presence of the incisal notch which is considered a weak point and contributes to midline fracture of the maxillary denture base [2]. Therefore, denture base reinforcement became mandatory to increase the resistance of the denture base to fracture by adding suitable reinforcing materials to the conventional acrylic denture during its fabrication. It is one of the several strategies aimed at improving denture base mechanical and physical properties, resulting in expanding the denture’s lifespan, minimizing fracture possibility, and consequently enhancing patient’s satisfaction [3].

Methods of denture reinforcement that have been enhanced and modified through the past decades include the addition of various materials, such as carbon fibers, glass fibers, and ultrahigh modulus polyethylene fibers [4]. Nevertheless, this method of denture reinforcement shows many drawbacks, such as the inability to balance biological, and /or mechanical properties effectively with lowering esthetic qualities, in addition to fiber fracture, debonding, and /or fiber pull-off [5, 6].

Metal frameworks were incorporated within the denture base to assist in producing complete dentures with better strength, volume stability, improved temperature sensation, high fracture resistance, and less deformation [7]. However, being a heavier denture with more complicated processing steps, in addition to bad esthetics, and sensitivity to metal alloys were considered the most common drawbacks of the metal-reinforced complete denture [8].

Recently, advanced ceramic materials such as zirconia have been introduced in an attempt to replace metal in dental restorations because of their improved mechanical properties, better appearance, absence of metallic color and taste, and finally, to enhance heat-polymerized complete denture base physical and mechanical properties [9].

With the evolution of digital technology, computer-aided design and computer-aided manufacturing (CAD/CAM) techniques have been introduced to many dental fields, aiming to simplify conventional fabrication steps with superior prosthesis quality in shorter processing time, besides overcoming the drawbacks of the conventional lost-wax processing technique [10–12].

Strain gauge analysis is considered to be a valuable tool used to measure the strain formed within an object when subjected to a certain amount and direction of forces and used to study stresses induced in dental structures. It is mainly used in in-vivo studies to evaluate the amount of strain induced on supporting structures [13]. Strain gauges can be used to investigate the biomechanical performance of different removable prosthesis materials, allowing their optimum choice according to the clinical situation [14].

Complete dentures gain their support from the oral mucosa and residual ridge [15], the variable materials used in denture base reinforcement could exert different amounts of pressure onto the tissue. Previous literature reviews revealed that reinforced maxillary denture bases have superior mechanical properties to conventional bases. On the other hand, little information is present regarding the amount of stress-transmitted under static and dynamic loading-to the oral supporting tissues and the degree of deformation that may occur to maxillary denture bases when reinforced with framework constructed digitally from either zirconia or cobalt chrome. Thus, this research is prompted to compare the effect of reinforcing maxillary complete dentures by using either zirconia or cobalt chrome frameworks constructed digitally by CAD/CAM technology. The null hypothesis was that no difference will be found in strains among the reinforced maxillary dentures having either zirconia or metal reinforcement frameworks.

## Materials and methods

The current study has been accepted by the committee of ethics (“in accordance with the Declaration of Helsinki”), Faculty of Dentistry, British University in Egypt with approval number (24–070).

### Denture grouping 

A total of twenty-six maxillary complete dentures were fabricated and divided into two groups (*n* = 13). Group A (test group): maxillary denture reinforced with zirconia framework. Group B (control group): maxillary denture reinforced with a Cobalt Chrome (Co-Cr) framework.

### Sample size calculation

A pilot study was conducted by unilaterally applying static loading on 5 samples of the zirconia and cobalt chrome (Co-Cr) groups then measuring the strain at the loaded area. The Cohen’s d effect size measured from the pilot study was 6.13. Based on the Cohen’s d effect size, with an assumed type I error of 0.05 and a study power of 0.9, using G Power software version 3.1.9.7. The sample size required to detect a significant difference between the two groups was increased to include 13 samples per group.

### Cast duplication

**Fig. 1:**
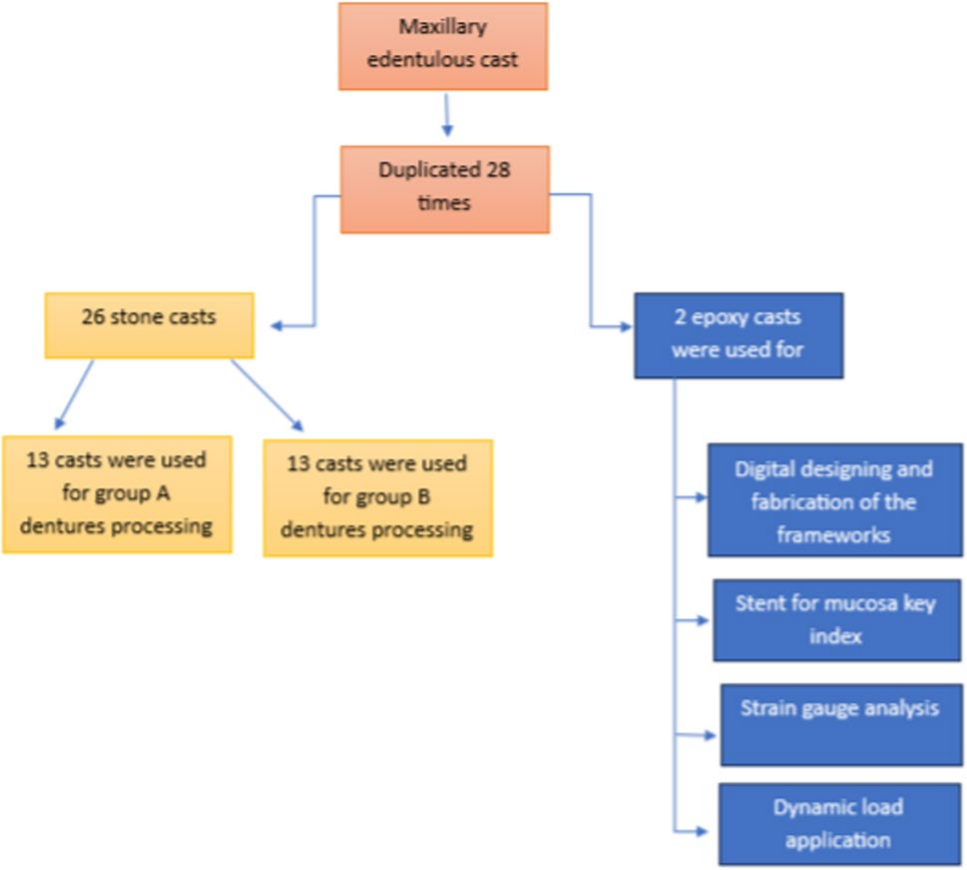
Flowchart summarizing the experimental workflow

An edentulous maxillary master educational cast was selected and duplicated 28 times using a silicon mold (Elite Double 8, Zhermack Group, Polesine (RO), Italy), 26 casts were poured with dental stone (type III stone, GH dental stone, Egypt) and used for dentures processing with reinforcement frameworks of both groups. The last two casts were poured from epoxy (RESIN Pro, Italy). These two epoxy casts-one cast for each group-were used for scanning and frameworks fabrication, production of stent for mucosa key index fabrication, strain gauge analysis, and dynamic chewing simulation (Fig. 1).

#### A- Framework designing and fabrication

**Fig. 2:**
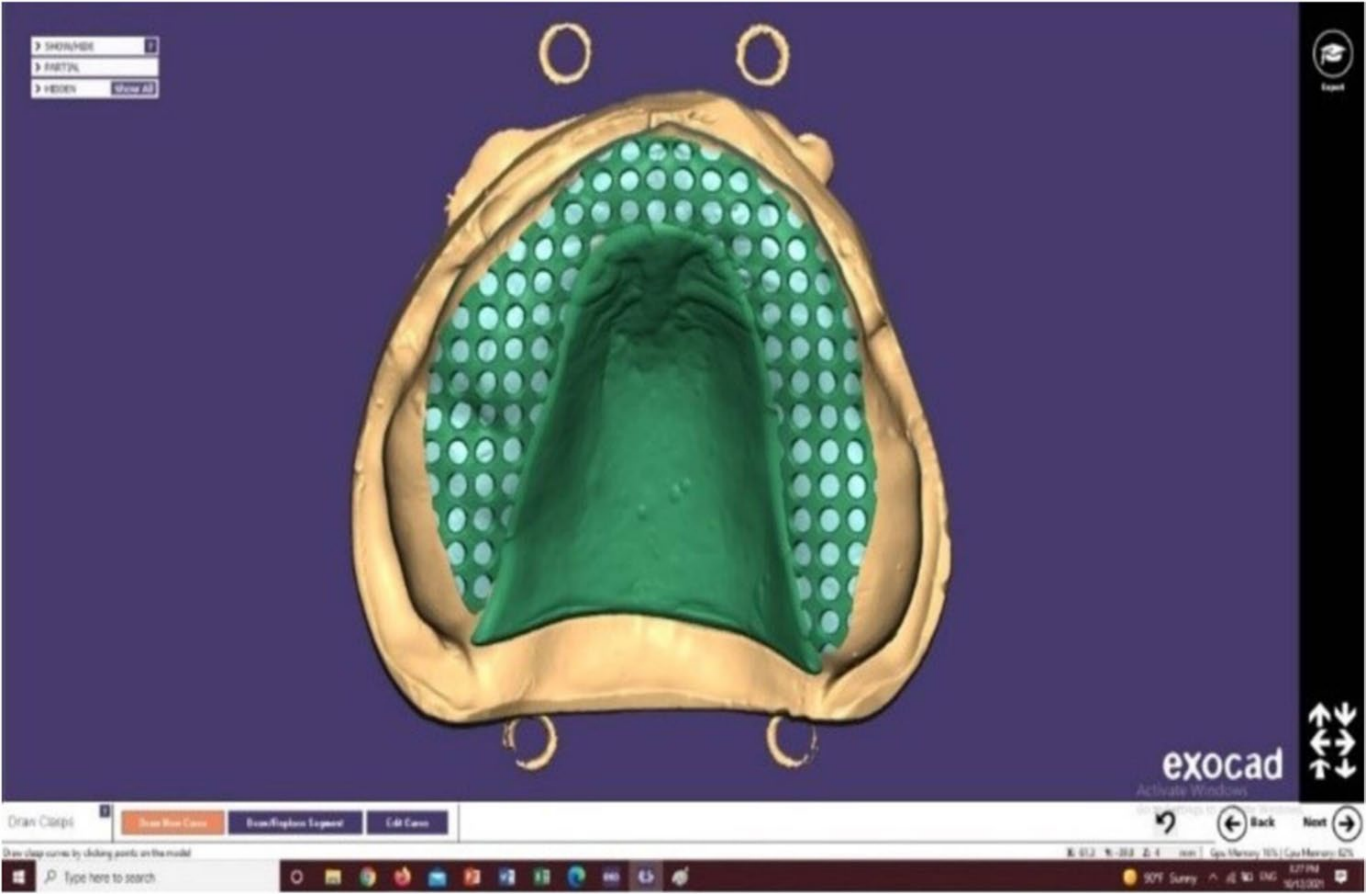
Digital framework design

The maxillary epoxy master cast was used for framework designing, first, beading was done underneath the place where the posterior end of the major connector was placed, extended from one hamular notch to another, its outline following the anatomy of the posterior palatal seal area and its depth was 0.5 mm, then, the cast was scanned using a desktop scanner (D850,3Shape, Copenhagen, Denmark) to get the standard triangulation file (STL). The STL file was then exported to Exocad software (PartialCAD,3.1; exocad GmbH) for digital framework designing. First, a proper relief of 1 mm thickness was created using the tissue stops icon over the edentulous ridge to accommodate for denture base thickness below the framework, then the selection of denture base design (meshwork) and a complete palatal plate major connector was done (Fig. 2).

**Fig. 3:**
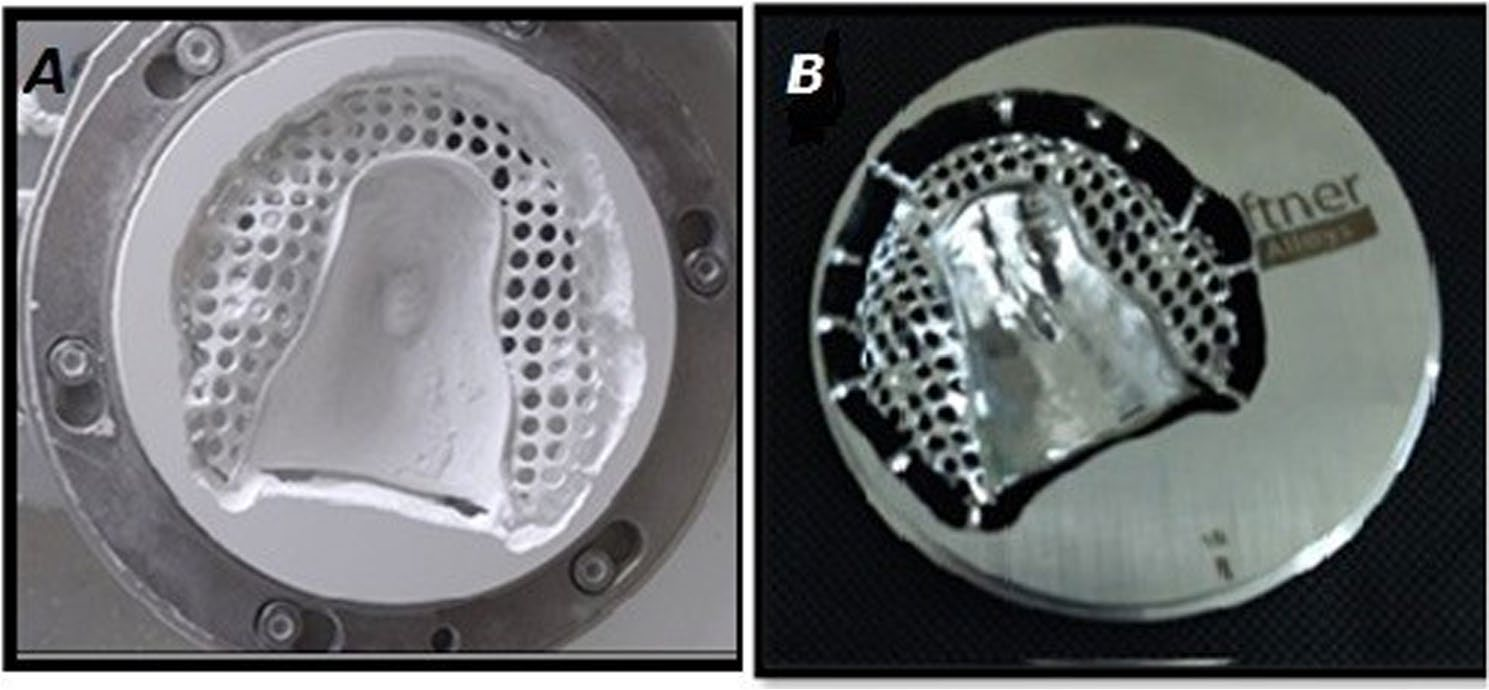
**A** Zirconia framework after milling, **B** Cobalt Chromium (Co-Cr) framework after milling

The framework with 1 mm thickness was then CAD/CAM milled with a 5-axis milling machine (CORiTEC 250i, imes-icore, Hesse, Germany) using zirconia discs (Nacera, Dental Zirconia Blank, Germany) of 98*25 mm dimensions, with a scale factor of 1.24. the zirconia discs were dry milled for an average milling time:300.23 min, complete sintering in the furnace at 1,350 ℃ to 1,500 ℃ was done. And using Cobalt chromium (Co-Cr) pre-sintered discs (MoguCera C Disc, Scheftner, Germany) of 98*18 mm dimensions were dry milled with an average milling time of 725.33 min (Fig. 3 A and B).

#### B- Maxillary denture fabrication

A waxed denture of about 3 mm denture base thickness (Cavex, Haarlem, Netherlands) was fabricated, artificial teeth (Acrostone, Egypt) were set according to the ideal teeth positions in relation to the ridge (palatal cusps of the posterior teeth were centered on the crest of the ridge following the lingualized occlusion concept, and then duplicated using a medium body rubber base Silicone (Elite Double 8, Zhermack Group, Polesine (RO), Italy) to create a silicon index of the outer and occlusal surfaces of the trial denture base was fabricated to standardize teeth size and arrangement with the rest of the denture bases in both groups.

#### C- Incorporation of the reinforcing frameworks into the maxillary denture

**Fig. 4:**
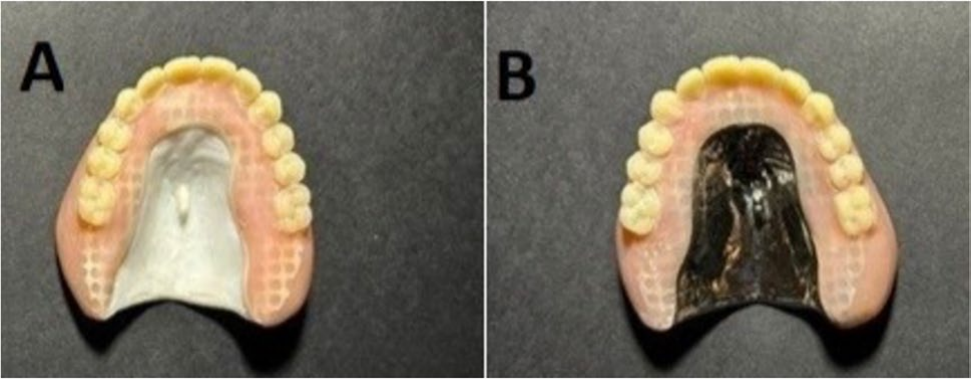
**A** Maxillary denture reinforced with zirconia framework. **B** Maxillary denture reinforced with cobalt chrome (co-cr) framework

The milled frameworks were located within the palatal part of the waxed denture, zirconia frameworks were treated by airborne-particle abrasion using alumina oxide particles (110 μm average diameter at 2.5 bar pressure), followed by ultrasonic cleaning in 99% isopropyl alcohol for 3 min before use [16], while Cobalt chromium (Co-Cr) frameworks were sandblasted by Aluminum oxide particles 250 μm at 3–4 bar pressure, followed by metal primer (Metal primer2, GC) for conditioning [17]. The waxed dentures were then invested and flasked using heat-polymerized polymethyl methacrylate (PMMA) acrylic resin (Acrostone, Egypt), which was packed carefully and processed conventionally according to the manufacturer’s instructions by a long curing cycle in a hot water bath at 72 0 C for 6.5 h [18], then deflasked, carefully examined for any obvious defects, finished, and polished (Fig. 4 A and B).

#### D- Simulation of mucosa covering the residual ridge

The epoxy casts were prepared to be used for strain gauge analysis and dynamic cyclic loading application. First the casts were coated with separating medium (Ainworth, Australia), and a negative model of the casts was made (mucosa key index) using dental stone (type III stone, GH dental stone, Egypt). Then, reduction was done to the edentulous cast surface using custom-made dental bur with a depth marker to create 2 mm of space for the artificial mucosa. A silicone material (Speedex addition silicon, Coltene, Switzerland) was placed on the reduced edentulous cast and covered with the negative model (mucosa key index) to fabricate the artificial mucosa. After complete setting of the silicone material, the mucosa key index was then removed, and the silicone layer was examined to be fully extended to cover the entire surface of the cast without any irregularities or tears. The main purpose of having a mucosa key index is to accurately replace the artificial mucosa if subjected to tears during force application [14, 19].

### Strain gauge analysis

It is the commonly used technique to evaluate the biomechanical loads exerted at specific areas under the denture base [20]. It consists of an insulating flexible backing that holds a metallic foil pattern attached to the object of interest by a suitable adhesive. As the object is deformed, the foil is deformed, changing its electrical resistance, and the maximum principal strain (MPS) is recorded [21].

Four strain gauges (Kyowa Electronic Instruments, Tokyo, Japan) with 1 mm length, 2.4 width, and 120 Ohm nominal resistance were cemented onto the cast surface using bonding agent (Colten, Switzerland) at the following areas: midline anterior (incisive papilla (IP), midline posterior (vibrating line (VL)), loaded area (right first molar area), and unloaded area (left first molar area.

### Adhesion site preparation for strain gauges cementation

An acrylic bur was used to prepare the selected areas by creating vertical slots designed according to the standard of strain gauge installation (2 mm width, 0.5 depth, and with a longitudinal axis parallel to the ridge long axis and perpendicular to the crest. The slot and surrounding area were cleaned with isopropyl alcohol (99.9%), the slot floor was lightly abraded with 320-grit alumina oxide sandpaper to create a microscopically rough surface, increasing surface area for bonding. This was followed by a second alcohol clean, and finally, a methyl methacrylate (MMA)-based primer (Visio-Bond) was applied to the prepared slot for the chemical conditioning of the cast surface. The primed surface was allowed to air-dry for 60 s as per manufacturer’s instructions.

### Adhesive type and curing protocol

- Adhesive Type
  Fast-cure, low-viscosity, flexible cyanoacrylate adhesive (M-Bond 200). This adhesive is used for strain gauge bonding to plastics and resins. Its low viscosity ensures a thin, uniform bond line (< 0.05 mm), and its flexibility minimizes the introduction of a local stiffening layer.
- Curing Protocol
  1. One drop of the adhesive was applied to the prepared slot.
  2. The strain gauges were mounted on their application tapes and were carefully adapted to the adhesive.
  3. A silicone rubber pad was placed over each gauge, and a uniform finger pressure was applied for 90 s—the manufacturer-specified time for initial fixture.
  4. The assembly was then left for 24 h at room temperature to achieve full bond strength and avoid dimensional changes before testing.

**Fig. 5:**
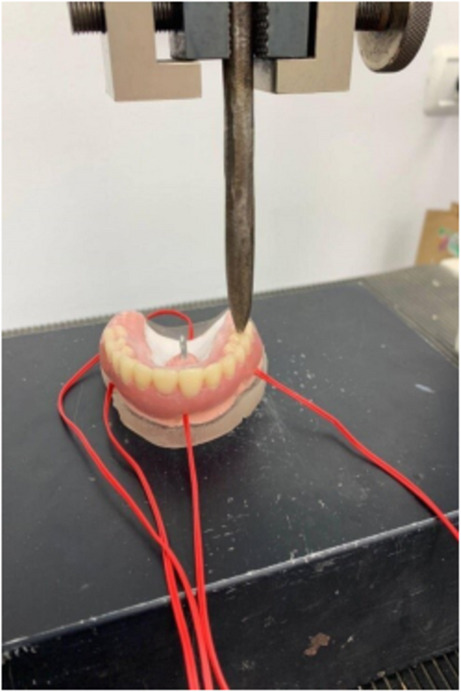
Unilateral vertical static load application for strain gauge analysis

The epoxy casts were secured on the universal testing machine with the occlusal plane placed parallel to the floor. A unilateral vertical static load of 200 N [22, 23] for one minute was applied by the universal testing machine (MMT250N, Shimadzu, Kyoto, Japan) through a special rod applicator with a rounded end at the right first molar central fossa (Fig. 5). The induced strain at the strain gauges was recorded through sensor interface boards. The process was repeated five times for each denture, and the average of the maximum principal strain (MPS) at each strain gauge was recorded and analyzed using a software program (PCD 300 A; Kyowa Electronic Instruments Co., Ltd.). The results were recorded, tabulated, and statistically analyzed.

### Scanning of the dentures and application of dynamic loading for deformation detection

All dentures’ fitting surfaces were scanned using a desktop scanner (3Shape E2) and exported as a standard tessellation language file (STL). The STL file was imported into the software and saved.

**Fig. 6:**
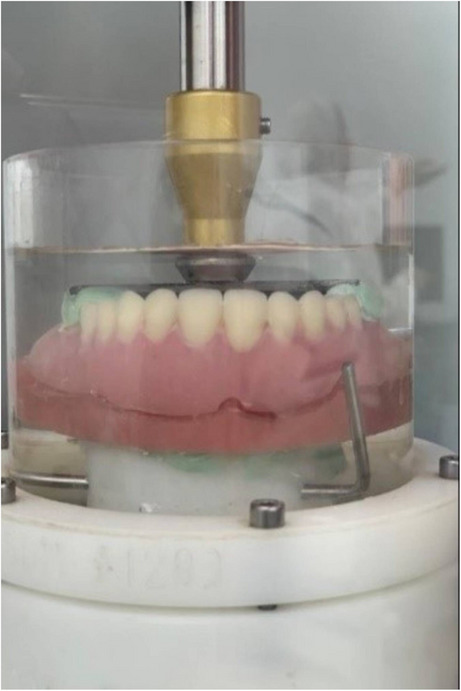
Reinforced denture bases inside the chewing simulator device

One reinforced denture from each group was seated on the epoxy resin cast with a metal plate that was attached previously to the occlusal surface of the denture in the center of the arch [24, 25], and introduced to the chewing simulator device (CS-44-SD Mechatronic chewing simulator, Munich Germany). A series of 300,000 (biaxial) cyclic loadings of 80 N, with 1 Hz frequency, 60 mm/sec speed, 3 mm vertical path, and 0.7 mm horizontal path [8], were conducted to simulate the maxillary denture function for one year using a stylus falling at the center of the metal plate (Fig. 6).

**Fig. 7:**
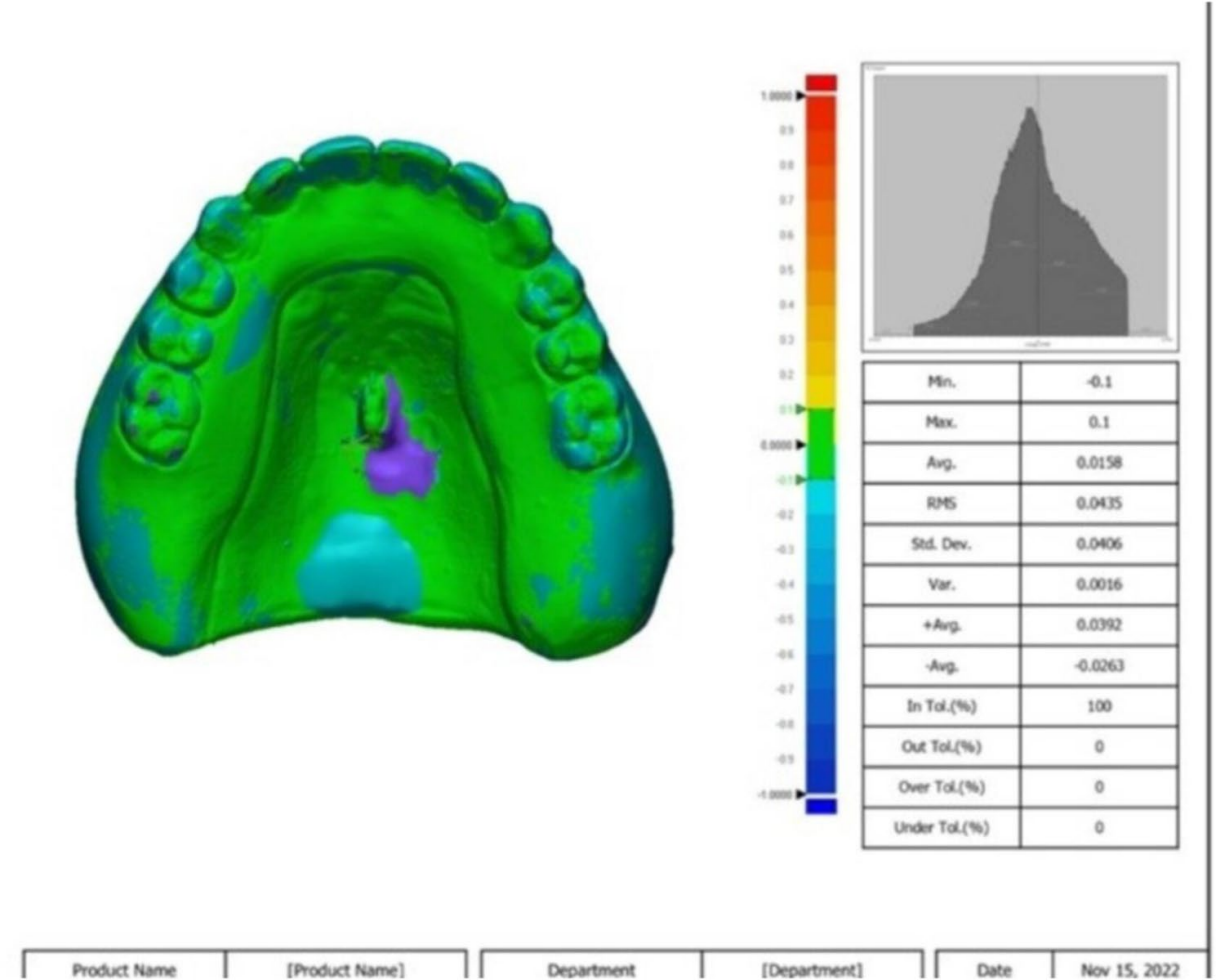
Geomagic report: Color map of fitting surface adaptation between the cast and the denture base after dynamic load application. Yellow to red indicates pressure from the denture fitting surface to the cast. Blue indicates space between the denture fitting surface and the cast. Green indicates that the denture fitting surface is in contact with the cast

After dynamic load application, the fitting surfaces of the dentures were scanned, and the STL file was imported to the software also to be superimposed to the previous STL file before dynamic load application using first initial alignment and then best-fit alignment in a 3-D measuring tool (Geomagic Control X, 3D Systems, United States) using surface matching software (Fig 7). Since each point’s measurements included both positive and negative values, the root mean square (RMS) (mm) was calculated.

### Statistical analysis

Data were explored for normality using histograms and Shapiro-Wilk test. Continuous data was presented as mean, standard deviation (SD), median and range (minimum - maximum) values. The Mann-Whitney U test was used for between-group comparisons.

The significance level for all tests was set at 0.05. Statistical analysis was performed using SPSS software (IBM Corp. Released 2017. IBM SPSS Statistics for Windows, Version 25.0. Armonk, NY: IBM Corp.)

## Results

### Strain gauge analysis

**Fig. 8:**
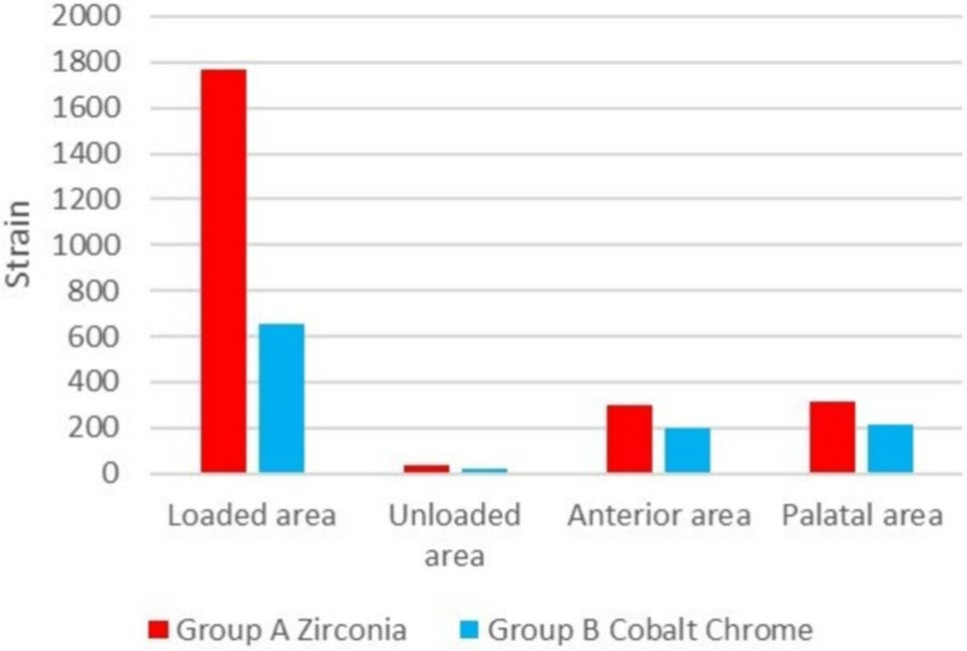
Bar chart representing the mean strain at different areas after static loading in both groups

After unilateral static loading, acrylic maxillary complete dentures reinforced with CAD/CAM milled zirconia framework (Group A) showed significantly higher strain measured at the four strain measuring areas compared to acrylic maxillary complete denture reinforced with CAD/CAM milled Cobalt Chrome (Co-Cr) framework (group B) (Fig. 8).

The maximum principal strain (MPS) at the loaded area (1st right molar area) was considered the highest among the measuring areas, followed by the palatal area, the anterior area, and the unloaded area, regardless of the denture reinforcement framework types.

### Deformation detection

**Fig. 9:**
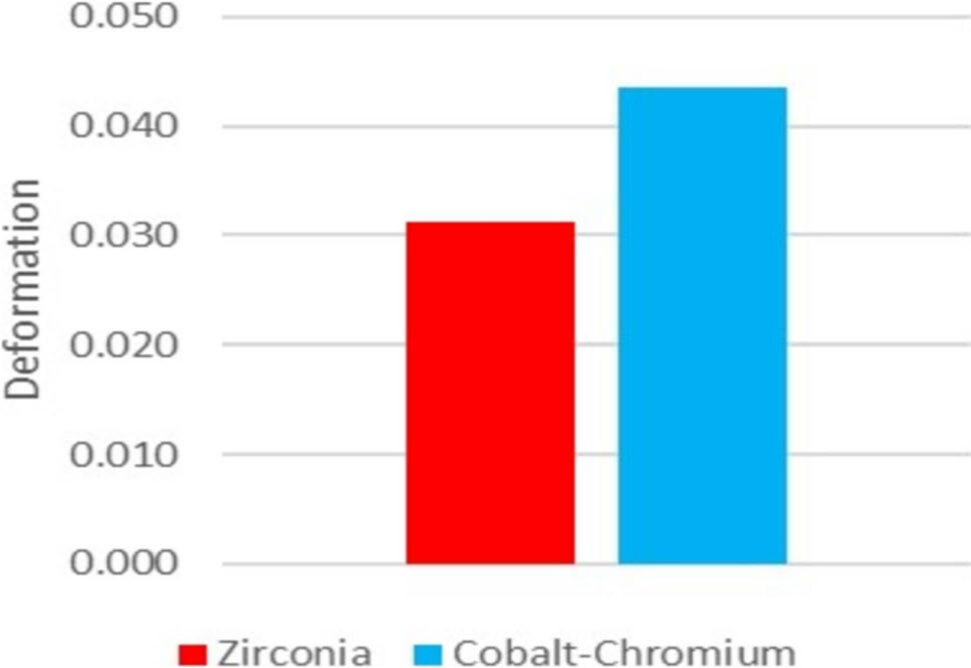
Bar chart representing the mean denture base deformation after dynamic cyclic loading in both groups

After dynamic cyclic load application, acrylic maxillary complete denture reinforced with CAD/CAM milled zirconia framework (Group A) showed statistically significant reduced deformation compared to acrylic maxillary complete denture reinforced with CAD/CAM milled cobalt chrome (Co-Cr) framework (group B) (Table 1) (Fig. 9).

**Table 1:** Mean, standard deviation, median and range values and the results of Mann – Whitney U test for comparison of strain at different areas and denture base deformation between the two groups

			Zirconia	Cobalt-Chromium	*p-*value
**After static loading**	**Strain at the loaded area**	**Mean (SD)**	1767.3 (231.9)	652.3 (111.4)	< 0.001
		**Median (Range)**	1765 (1405–2105)	695 (425–770)	
	**Strain at the unloaded area**	**Mean (SD)**	32.3 (3.3)	19.2 (1.9)	< 0.001
		**Median (Range)**	30 (30–40)	20 (15–20)	
	**Strain at the anterior area (IP)**	**Mean (SD)**	297.7 (28.8)	197.3 (36.7)	< 0.001
		**Median (Range)**	310 (230–320)	190 (145–255)	
	**Strain at the palatal area (VL)**	**Mean (SD)**	313.8 (20.2)	211.9 (29.3)	< 0.001
		**Median (Range)**	315 (280–345)	225 (160–250)	
**After cyclic loading**	**Denture base deformation**	**Mean (SD)**	0.031 (0.005)	0.044 (0.005)	< 0.001
		**Median (Range)**	0.032 (0.024–0.039)	0.043 (0.036–0.052)	

There were differences in strain induced to the supporting tissues and deformation detected between Co-Cr and zirconia frameworks. Thus, the null hypothesis was rejected. The results elaborated that maxillary dentures reinforced with a zirconia framework showed less deformation but transmitted more strain to the supporting structures compared to maxillary complete dentures reinforced with a cobalt chrome framework at all four strain measuring areas (loading, non-loading, anterior midline, and posterior midline). It was found that the highest mean of micro strain values were recorded by the strain gauges located at the loading area was 1767.3 (231.9) followed by the posterior midline area 313.8 (20.2), the anterior midline area 297.7 (28.8), while the lowest mean of micro strains were recorded at the non-loading area 32.3 (3.3), while maxillary dentures reinforced with cobalt chrome framework mean values of micro strain at the loading area was 652.3 (111.4), at the posterior midline area was 211.9 (29.3), at the anterior midline area was 197.3 (36.7), while at the non-loading area was 19.2 (1.9).

## Discussion

Preserving the supporting tissues underneath the denture is one of the most crucial factors that influence the longevity of any dental prosthesis. Numerous tests were conducted to ensure that the stresses are evenly distributed during mastication and the resultant strain created within the supporting tissues is favorable, and safe [26].

In the present study, a non-reinforced denture base group wasn’t needed, as it is well known that, regardless of the framework material used, the reinforced dentures are expected to withstand more force and show less deformation compared to the non-reinforced ones. This was also proven by Amaral, et al. The authors declared that better distribution of stress with less denture base fracture was reported with the co-cr framework-reinforced mandibular overdenture compared to the non-reinforced ones [27].

It is well known that fabricating reinforced frameworks using a traditional processing technique, such as casting, would necessitate complex and expert skills. Conversely, building the framework with CAD/CAM systems is easy, doesn’t call for complex methods or a high level of expertise, and allows for the option of different materials. Patients can have complete dentures that are lighter, more comfortable, more durable, and thinner if the right material is used for the palatal region. Along with all the benefits of this technology, such as faster production, improved wear characteristics, reduced time and effort spent in the lab, and better control over cross-infection, CAD-CAM was utilized to guarantee a precisely planned and machined framework [28, 29].

Fracture is liable to occur with metal-reinforced dentures as a result of poor adhesion between PMMA and cobalt chrome, to avoid this, surface treatment to the metal framework including sandblasting and metal primer application, was found useful to enhance fracture resistance [21].

Since variations in the thickness of mucosa can either raise or reduce the stress imparted to the supporting tissues, a mold with a constant thickness of 2 mm is used to manufacture the mucosa key index. As previously noted, the underlying soft tissue has an impact on denture foundation deformation [30].

A digital loading device was utilized to apply a static loading occlusally with a vertical load of 200 N on the central fossa of the first molar crown. Several earlier investigations used the same force magnitude and direction [22, 23, 31].

The static load was applied at the posterior region of the complete denture unilaterally. This situation is similar to the force applied to a complete denture during chewing.

It is preferable to bond the strain gauge on a completely flat surface to minimize the possibility of obtaining incremental apparent strain that results from mounting the strain gauge on a curved surface; therefore, the strain gauges were installed on prepared flat surfaces parallel to the ridge’s long axis and perpendicular to its crest [32].

Using a stylus that fell in the middle of the metal plate that had previously been fixed to the maxillary complete denture’s occlusal surface, the chewing simulator applied a dynamic cyclic loading. The metal plate was selected over an acrylic one because prior research had shown that it had a better force distribution [9].

The null hypothesis, whereby the difference in reinforcement framework material will not affect the strain found under the maxillary dentures, was rejected. According to the literature, the modulus of elasticity of a material is a key factor that directly affects the amount of stress absorbed by the material and its manner of distribution through the material to the underlying structures [33, 34].

The overall strains developed under maxillary complete dentures reinforced with zirconia frameworks were statistically higher than those produced under maxillary complete dentures reinforced by cobalt chrome frameworks when vertical load was applied. This could be attributed to the mechanical properties of the cobalt chrome which showed a higher modulus of elasticity (210–253 GPa) [35, 36] than zirconia (92-209.3 GPa). Besides, zirconia is a tough material that couldn’t absorb the applied forces; instead, it caused high stress concentration to the underlying supporting tissues rather than being resilient, flexing, and absorbing the applied force and transferring less pressure to the underlying supporting oral tissues as the cobalt chromium framework did [37–39].

This was in accordance with a study focused on the mechanical properties of mandibular implant overdentures with reinforced zirconium meshwork bases and compared them to cobalt chromium meshwork-reinforced bases; it was found that mandibular overdentures reinforced by cobalt chrome meshwork showed higher fracture resistance under fatigue cyclic loading [40]. That means that zirconia is more brittle.

Another study was done by Hada T et al. to detect maxillary denture deformation reinforced with CAD/CAM milled different framework materials (nano-zirconia, cobalt chrome, PEEK, PMMA, and fiber reinforcement composite). It was observed that the nano-zirconia framework showed smaller deformation compared to the cobalt chrome framework [19], the same as found in this study.

Additionally, when comparing cobalt chrome frameworks with PEEK ones regarding stresses transmitted to the supporting structures, it was found that mandibular complete dentures reinforced by PEEK frameworks transmit higher loads than cobalt chromium ones in all strain loading areas. It might be due to the fact that, cobalt chrome alloys showed higher tensile strength and modulus of elasticity than PEEK material [34, 41].

These findings were following many other studies that compared materials, such as PEEK and Bio-HPP frameworks, with cobalt chrome frameworks, and they revealed that cobalt chrome induces lower stresses to the supporting structures when load was applied, whether this load was unilateral or bilateral, due to its resiliency that could dissipate the applied forces with its high rigidity that enables wide distribution of load and decreases load per unit area [9, 14, 38].

Regarding deformation recorded after dynamic cyclic loading, it was found that group 1 maxillary dentures reinforced with zirconia framework showed less deformation than maxillary dentures reinforced with cobalt chrome framework; this could also be related to their mechanical properties, considering that zirconia is a brittle material, so crack propagation and fracture are its standard behaviors under loading; however, cobalt chrome is considered as a shock absorber that can dissipate the applied load within the material instead of transmitting it to the underlying supporting tissues, causing more deformation to the framework. These findings were confirmed by a study that detected deformation occurring in cobalt chrome clasps compared to acetal resin clasps after cyclic loading for a 2-year period and which found that cobalt chrome clasps exhibit significantly more deformation than acetal resin clasps [42].

However, another study the deformation of a maxillary complete denture by comparing the effects of various denture strengtheners, such as wires (made of metal) and rods (made of fiberglass), on the deformation of a maxillary complete denture. It revealed that the deformation of the denture was reduced by about 53–56% compared to the non-reinforced dentures. The study claimed that the metal wire-reinforced dentures showed no significant differences in maximum principal strain (MPS) values compared to those of fiberglass-reinforced dentures [23], which was not found in our study.

Zhang et al. [43] studied the fatigue behavior of zirconia after 5 million fatigue cycles, and showed that zirconia exhibited more fractures after cyclic loading, leading to the recommendation to use it in the anterior rather than the posterior areas of the dental arch. However, in a similar study on the fatigue of zirconia under cyclic loading, Studart et al. found that crack propagation in zirconia frameworks was significantly subcritical, and explained that the high mechanical properties of the zirconia frameworks were reflected positively on their lifetime could be extended to more than 20 years of clinical performance [44].

### Limitations of the study

- Strain gauge analysis is limited to in vitro studies and can’t be used intraorally.
- The material of artificial teeth may affect the amount of stain created under the denture base and the distribution of the applied forces.

### Recommendation for future studies

- The present study examined the strain under only simple conditions of occlusal force application. However, in clinical situations, denture fractures have always occurred as a result of fatigue failures; therefore, it is thought that a fatigue test is especially important in such analyses. Future studies are therefore necessary to determine the fatigue behavior of such reinforced dentures.
- Also, the effect of posterior teeth material and arrangement on complete denture deformation may be a topic of future research.

## Conclusion

- Within the limitations of this in vitro study, the laboratory evaluation using micro-strain recording revealed that zirconia framework-reinforced maxillary dentures were associated with higher strain developed to the supporting tissues but with a lesser degree of deformation than cobalt chrome framework-reinforced maxillary dentures when static and dynamic loads were applied.
- Both maxillary dentures reinforced with CAD/CAM-milled zirconia or cobalt chrome frameworks exhibited a sufficient reinforcement effect against complete denture deformation.
- In clinical conditions with flat or flabby ridges, it is recommended to use cobalt chrome framework-reinforced maxillary dentures; however, zirconia framework-reinforced maxillary dentures are more advisable to be used with a well-formed ridge covered with healthy mucosa.

## Data Availability

There are no data underlying the manuscript; all data were presented by figures and tables in the current study.

## Abbreviations

CAD/CAM: Computer-Aided Designing/Computer-Aided Manufacturing
MPS: Maximum principal strain
Co-Cr: Cobalt Chrome
IP: Incisive papilla
VL: Vibrating line
